# Genome Sequence of the 2,4,5-Trichlorophenoxyacetate-Degrading Bacterium *Burkholderia phenoliruptrix* Strain AC1100

**DOI:** 10.1128/genomeA.00600-13

**Published:** 2013-08-08

**Authors:** Ping Xu, Hao Yu, Ananda M. Chakrabarty, Luying Xun

**Affiliations:** School of Molecular Biosciences, Washington State University, Pullman, Washington, USAa; State Key Laboratory of Microbial Metabolism, Shanghai Jiao Tong University, Shanghai, Chinab; Department of Physiology and Biophysics, University of Illinois College of Medicine, Chicago, Illinois, USAc

## Abstract

*Burkholderia phenoliruptrix* strain AC1100 (ATCC 53867) degrades a variety of recalcitrant xenobiotics, including 2,4,5-trichlorophenoxyacetate. The molecular mechanism of 2,4,5-trichlorophenoxyacetate degradation has been extensively studied. Here we present a 7.8-Mb assembly of the genome sequence of this 2,4,5-trichlorophenoxyacetate-degrading strain, which may provide useful information related to the degradation of chlorinated aromatic compounds.

## GENOME ANNOUNCEMENT

Strains of the genus *Burkholderia* occupy a wide range of ecological niches and have versatile properties of bioremediation, biocontrol, and plant growth promotion (1). The herbicide 2,4,5-trichlorophenoxyacetic acid (2,4,5-T), a suspected carcinogen, is a component of Agent Orange that was used in the Vietnam War and created long-term health problems (2, 3). *Burkholderia phenoliruptrix* AC1100 (ATCC 53867) (formerly *Pseudomonas cepacia* AC1100 and *Burkholderia cepacia* AC1100) was the first pure culture that used 2,4,5-trichlorophenoxyacetate as a sole source of carbon and energy (4–6). Three gene clusters, *tftAB*, *tftCD*, and *tftEFGH*, are involved in converting 2,4,5-trichlorophenoxyacetate to 3-oxoadipate in *B. phenoliruptrix* AC1100 (6–10). An IS element adjacent to the *tftAB* gene cluster provides the promoter for *tftAB*. The 2,4,5-trichlorophenoxyacetate-degrading ability is unstable due to the loss of the promoter created by the IS element (6, 11, 12). Thus, 2,4,5-trichlorophenoxyacetate is usually used as the sole carbon and energy source to cultivate *B. phenoliruptrix* AC1100 to preserve its ability for 2,4,5-trichlorophenoxyacetate degradation. Here, we present the genome sequence of *B*. *phenoliruptrix* AC1100, providing genomic contents for the biodegradation of 2,4,5-trichlorophenoxyacetate and other chlorinated aromatic compounds.

The genome sequence of *B. phenoliruptrix* AC1100 was obtained using the Illumina HiSeq 2000 system (100-bp paired-end sequencing). The reads were *de novo* assembled with Velvet software to 286 contigs (>500 bp), providing 44-fold coverage (13). The contig *N*_50_ was 81,158 bp, and the largest contig was 305,883 bp. Gene prediction and genome annotation were performed using of the RAST autoannotation server and NCBI PAPPC pipeline (14, 15). The tRNA genes were predicted using tRNAscan software (16). The gene function and classification were performed using the KEGG and Clusters of orthologous Groups (COG) databases (17).

The draft genome sequence of strain AC1100 comprises 7,811,030 bp, with a G+C content of 63.1%. There are 7,443 predicted protein coding sequences (CDS) (877 bp average length, 83.5% coding density). The genome of strain AC1100 has 1 rRNA operon and 52 tRNA loci. There are 493 subsystems represented in the genome sequence (2,844 CDS in total), and the metabolic network of AC1100 (determined by the RAST server) was reconstructed (14). We have predicted a rich set of genes (189 CDS) responsible for the degradation of aromatic compounds and 176 CDS for stress responses. Operons of *tftAB*, *tftCD*, and *tftEFGH* were found located on three contigs, and characteristics of the sequence are different from the core genome, indicating that they might belong to mobile regions of the genome (4–6). The genes for the degradation of catechol (catechol 1,2-dioxygenase), benzoate (benzoate 1,2-dioxygenase and benzoate-4-monooxygenase), toluene (toluene 4-monooxygenase), and the 3-oxoadipate pathway, homogentisate pathway, and central meta-cleavage pathway were predicted. The bacterium may have powerful degradation potentials for aromatic compounds. The genomic information of strain AC1100 will provide new insights into the genetic versatility of *Burkholderia* species and the metabolism of complex aromatic compounds.

### Nucleotide sequence accession numbers.

This whole-genome shotgun project has been deposited at DDBJ/EMBL/GenBank under the accession number ASXI00000000. The version described in this paper is the first version, ASXI01000000.
